# Vitamin D receptor expression is essential during retinal vascular development and attenuation of neovascularization by 1, 25(OH)_2_D_3_

**DOI:** 10.1371/journal.pone.0190131

**Published:** 2017-12-22

**Authors:** Nasim Jamali, Shoujian Wang, Soesiawati R. Darjatmoko, Christine M. Sorenson, Nader Sheibani

**Affiliations:** 1 Departments of Ophthalmology and Visual Sciences, University of Wisconsin School of Medicine and Public Health, Madison, WI, United States of America; 2 McPherson Eye Research Institute, University of Wisconsin School of Medicine and Public Health, Madison, WI, United States of America; 3 Department of Pediatrics, University of Wisconsin School of Medicine and Public Health, Madison, WI, United States of America; 4 Department of Cell and Regenerative Biology, University of Wisconsin School of Medicine and Public Health, Madison, WI, United States of America; 5 Department of Biomedical Engineering, University of Wisconsin, Madison, WI, United States of America; University of Alabama at Birmingham, UNITED STATES

## Abstract

Vitamin D provides a significant benefit to human health, and its deficiency has been linked to a variety of diseases including cancer. Vitamin D exhibits anticancer effects perhaps through inhibition of angiogenesis. We previously showed that the active form of vitamin D (1, 25(OH)_2_D_3_; calcitriol) is a potent inhibitor of angiogenesis in mouse model of oxygen-induced ischemic retinopathy (OIR). Many of vitamin D’s actions are mediated through vitamin D receptor (VDR). However, the role VDR expression plays in vascular development and inhibition of neovascularization by 1, 25(OH)_2_D_3_ remains unknown. Here using wild type (*Vdr* +/+) and *Vdr*-deficient (*Vdr* -/-) mice, we determined the impact of *Vdr* expression on postnatal development of retinal vasculature and retinal neovascularization during OIR. We observed no significant effect on postnatal retinal vascular development in *Vdr* -/- mice up to postnatal day 21 (P21) compared with *Vdr* +/+ mice. However, we observed an increase in density of pericytes (PC) and a decrease in density of endothelial cells (EC) in P42 *Vdr* -/- mice compared with *Vdr* +/+ mice, resulting in a significant decrease in the EC/PC ratio. Although we observed no significant impact on vessel obliteration and retinal neovascularization in *Vdr* -/- mice compared with *Vdr* +/+ mice during OIR, the VDR expression was essential for inhibition of retinal neovascularization by 1, 25(OH)_2_D_3_. In addition, the adverse impact of 1, 25(OH)_2_D_3_ treatment on the mouse bodyweight was also dependent on VDR expression. Thus, VDR expression plays a significant role during retinal vascular development, especially during maturation of retinal vasculature by promoting PC quiescence and EC survival, and inhibition of ischemia-mediated retinal neovascularization by 1, 25(OH)_2_D_3_.

## Introduction

Vitamin D Receptor (VDR) is a member of the nuclear transcription factor superfamily. Through activation by vitamin D, VDR could associated with other nuclear transcription factors including retinoid-X-receptor (RXRα) and binds to the vitamin D response element in target genes causing expression or transrepression [1, 2]. The majority of vitamin D action is believed to be mediated through VDR. Genetic variation in VDR could lead to vitamin D deficiency, which is associated with increased risk for cancer and a variety of other diseases. VDR is detectable in almost all human tissues. In the eye, VDR is detected in retinal ganglion cell layer, inner nuclear layer, retinal pigment epithelium and the epithelium of cornea, lens, ciliary body, and retinal photoreceptor cells [3, 4].

The expression of VDR in tissues that are not traditionally involved in calcium metabolism, emphasizes a potential important role for vitamin D and its receptor in function of these tissues. Recently, a narrative review suggested the ability of eye tissue to locally produce vitamin D [3]. We recently assessed VDR expression in cells isolated from the retinal vasculature. Retinal pericytes (PC) express a high level of VDR compared to endothelial cells (EC), and its levels increased significantly by incubation of these cells with 1, 25(OH)_2_D_3_, the active form of vitamin D [5]. Therefore, investigating the role of vitamin D and its receptor in developmental processes and cell autonomous functions will help to better understand mechanisms of vitamin D action in various tissues including the eye.

The mouse retinal vasculature develops after birth, and provides a great opportunity to study all aspects of vascular development postnatally. Mice are born without retinal blood vessels. During the first week of life, the blood vessels sprout radially from the optic nerve to the edge of the retina forming the superficial layer of retinal blood vessels. These vessels then sprout deep into the retina and form the deep and intermediate layer of retinal vasculature, respectively. Formation of all vascular layers are complete by three weeks of age (postnatal day 21; P21). These vessels continue undergoing pruning, remodeling, and maturation, which is completed by 6 weeks of age (P42) [6–9]. The role VDR expression plays in retinal vascular development has not been previously addressed.

Retinopathy of prematurity (ROP) is a leading cause of blindness in premature infants (14%) [10, 11]. In the United States, about 15,000 premature infants develop some degree of ROP every year, and about 500 of them become legally blind due to severity of ROP [12]. In premature infants, incomplete vascularized retina and cycles of hyper- and hypo- oxygenation lead to formation of abnormal new blood vessels. These vessels grow from the retina into the vitreous and cause hemorrhage, and retinal detachment if left untreated. Thus, there is a great interest in understanding the underlying mechanisms responsible for sensitivity of developing retinal vasculature to high oxygen and development of therapeutics that save vision.

The mouse oxygen-induced ischemic retinopathy (OIR) is a highly reproducible model for study of retinal neovascularization [13], which recapitulates hyperoxia damage to the developing retinal vasculature observed in premature infants with ROP. In the OIR model, P7 pups and their mother are exposed to high oxygen (75%) for 5 days. This level of oxygen prevents further development of retinal vasculature and causes vessel loss around the optic nerve (vaso-obliteration). The mice are then brought to room air (20% oxygen) for 5 days. Lack of sufficient oxygen leads to neovascularization and abnormal growth of retinal blood vessels from the retina into the vitreous. Maximum retinal neovascularization occurs by P17 when mice are sacrificed for quantitative assessment of neovascularization. Our previous studies demonstrated that 1, 25(OH)_2_D_3_ inhibits retinal neovascularization during OIR [14]. However, the role VDR expression plays in retinal neovascularization and its inhibition by 1, 25(OH)_2_D_3_ during OIR remain unknown.

Here to investigate the role of VDR expression in vascular development and neovascularization, we compared normal postnatal retinal vascular development and retinal neovascularization during OIR in wild type (*Vdr* +/+) and *Vdr-*deficient *(Vdr* -/-) mice. Our results demonstrated that normal retinal vascular development is independent of VDR expression before weaning. However, the density and integrity of retinal vasculature was impacted during the late stages of development and maturation in *Vdr* -/- mice. The ratio of EC to PC decreased significantly, due to increased number of PC and decreased number of EC in P42 *Vdr* -/- mice. In addition, the degree of retinal neovascularization during OIR was independent of VDR expression. However, 1, 25(OH)_2_D_3_ failed to inhibit retinal neovascularization during OIR in *Vdr* -/- mice, unlike *Vdr* +/+ mice, indicating VDR expression is required for significant inhibition of neovascularization by 1, 25(OH)_2_D_3_. Interestingly, weight loss associated with 1, 25(OH)_2_D_3_ administration was not observed in *Vdr* -/- mice as occurred in *Vdr* +/+ mice. Thus, the adverse systemic effect of 1, 25(OH)_2_D_3_ on bodyweight is also dependent on VDR expression.

## Materials and methods

### Ethics statement

All animal experiments were performed in accordance to the Association for Research in Vision and Ophthalmology Statement for the Use of Animals in Ophthalmic and Vision Research and were approved by the Institutional Animal Care and Use Committee of the University of Wisconsin School of Medicine and Public Health (the assurance number A3368-01). Animals were sacrificed according to an approved protocol by CO2 asphyxiation.

### Animals

The vitamin D receptor (*Vdr*)-deficient mice (B6.129S4-*Vdr*^*tm1Mbd*^/J; Jackson Laboratories, Bar Harbor, ME; stock number 006133) and wild type mice were maintained at the University of Wisconsin animal facilities according to approved protocols. Litters were produced by mating heterozygote mutant mice and they were genotyped by PCR of DNA extracted from tail tips. The screening primers used for genotyping were as follows (5’ to 3’): *Vdr* mutant: CACGAGACTAGTGAGACGTG; *Vdr* wild type: CTCCATCCCCATGTGTCTTT; and *Vdr* common: TTCTTCAGTGGCCAGCT CTT, as suggested by the supplier. The impact of OIR on retinal vessel obliteration and neovascularization is sex independent [13]. We also confirmed this by assessing the degree of neovascularization and vessel obliteration in wild type male and female mice (C57BL/6j; Jackson Laboratories), and both responded similarly. Clinical studies also showed independence of sex with ROP as a risk factor [15, 16]. In all experiments male and female *Vdr* -/- mice were compared to their *Vdr* +/- and *Vdr* +/+ littermates. C57BL/6j mice (Jackson Laboratories) were also used as wild type control in some experiments.

For the mouse model of OIR, P7 pups and their mother were exposed to the hyperoxia condition (75% ± 0.5% oxygen) in an airtight incubator for 5 days as previously described [13, 17, 18]. Incubator temperature was maintained at 23 ± 2°C and oxygen level was monitored continuously using a PROOX model 110 oxygen controller (Reming Bioinstruments Co., Redfield, NY). The mice were then brought to room air (hypoxia, 20% oxygen) for 5 days. To investigate antiangiogenic activity of 1,25(OH)_2_D_3_, half of the pups from *Vdr* +/+ and *Vdr* -/- mice received intraperitoneal injection of 0.0125 μg (2.5 μg/kg) of 1,25(OH)_2_D_3_ (NDC 17478-931-01; Akorn, Inc.,Lake Forest, IL) from P12 to P16. The other half of the pups received saline alone. Mice were then sacrificed at P17, and one eye was collected for retinal wholemount preparation as described below and one eye was prepared for histological evaluations.

### Preparation, visualization, and analysis of retinal wholemounts

Mice were sacrificed at various time points during development. The mice eyes were enucleated and fixed in 4% paraformaldehyde briefly (3–5 min), then transferred to 70% methanol to fix for at least 24 h at -20°C. Retinas were dissected in and then washed with phosphate buffered saline (PBS) three times, 10 min each. Dissected retinas were then incubated in blocking buffer (50% fetal calf serum (FBS) and 20% normal goat serum (NGS) in PBS) for 2 h at room temperature (RT). Retinas were incubated with desired primary antibodies including rabbit anti-mouse collagen IV (Millipore, AB756P), anti-glial fibrillary acid protein (GFAP) (14-9892-82; eBioscience, ThermoFisher, San Diego, CA), and FITC- conjugated anti-α-smooth muscle actin (F3777; Sigma-Aldrich, St. Louis, MO) diluted 1:500 in PBS containing 20% FCS, 20% NGS at 4°C overnight. Retinas were then washed three times with PBS, 10 min each; incubated with appropriate secondary antibodies including Cy^TM^ 2-conjugated goat-anti-mouse (115-225-146; Jackson ImmunoResearch Laboratories, West Grove, PA) and Alexa 594 goat-anti-rabbit (A-11037; ThermoFisher) diluted 1:500 in PBS containing 20% FCS, 20% NGS for 2 h at RT. Retinas were then washed four times with PBS, 10 min each and mounted on the slide with PBS/glycerol (1:1 vol).

To assess the number of angiogenic sprouts at postnatal day 5 (P5), number of leading sprouts at the edge of expanding retinal vasculature were counted for at least six images per retina (at x100). Percentage of retinal vascular coverage was calculated by ratio of measured area of the expanded retinal vasculature to the total retina at P5.

To evaluate organization of major blood vessels, number of major arteries branched off of optic nerve were counted at P7 and P21 in images captured from anti- α-SMA stained retinas at x12.5. Development of vascular plexus were assessed by counting the number of immediate secondary branches and their associated branch points from the major arteries in the above images at both P7 and P21.

To assess the number of proliferating cells, Ki-67 staining was performed after completion of 2 h blocking. Briefly, retinas were incubated with Ki-67 (12075S; Cell Signaling) prepared (1:100) in 3% BSA, 0.3% Triton X-100 in PBS overnight at 4°C. The next day, retinas were washed three times with PBS, 10 min each. The retinas were then incubated with Isolectin B4-FITC (1:100; Vector Labs, Burlingame, CA) for 2 h and washed four times with PBS, 15 min each. The samples were mounted with PBS/glycerol (1:1 vol). For quantification, the mean number of Ki-67 positive cells on the intermediate layer of retinal vasculature were determined per field (x400). Retinas were viewed by fluorescence microscopy and images were captured using EVOS imaging system (AMG, ThermoFisher) or Zeiss microscope (AxioPhot, Carl Zeiss, Chester, VA).

### Quantification of retinal neovascularization and avascular area

Quantification of retinal and vitreous neovascularization during OIR (at P17) was performed using serial histological sections and image analysis as described previously by others [13, 19] and us [7, 17]. For histology sections, mouse eye were enucleated, fixed in formalin for at least 24 h at room temperature. They were then embedded in paraffin and eight serial sections (40 μm apart, 6 μm thick) were taken from around the optic nerve (four on each side of the optic nerve). The hematoxylin and PAS stained slides were examined for the presence of neovascular tufts grown from the retina into the vitreous, and the average from 8 sections is reported as the mean number of neovascular nuclei per eye.

For image analysis, after wholemount staining and imaging, retinal neovascularization was assessed by semi-automated quantification method (SWIFT_NV) installed on ImageJ software (NIH, Maryland, USA) as developed and described by Stalh et.al. [19]. During the preparation of digital captured images for these macros, avascular areas were also measured and reported as percentage of vaso-obliterated area relative to the total retina area.

### Immunohistochemical staining of the frozen sections

Enucleated mouse eyes were embedded in optimal cutting temperature (OCT) compound and frozen sections (6 sections per eye) were prepared. Sections were fixed in cold acetone for 10 min, washed three times with PBS (5 min each), and blocked (1% BSA, 0.2% skim milk, and 0.3% Triton X-100 in PBS) for 15 min at RT. Sections were then incubated with rabbit anti-mouse collagen IV (AB756P; Millipore) diluted in blocking solution (1:500) overnight at 4°C in a humid environment. The next day, sections were washed three times (5 min each) in PBS and incubated with secondary antibody Alexa 594 goat-anti-rabbit (1:500, prepared in blocking solution) (A-11037, Invitrogen). After three PBS washes, sections covered with PBS/glycerol (1:1 vol) and mounted with coverslip. Retinas were viewed using fluorescence microscopy and images were captured in digital format using a Zeiss microscope (Cal Zeiss, Chester, VA).

### Trypsin-digested retinal vessel preparations

Enucleated eyes from P21 and P42 mice were fixed in 4% paraformaldehyde for at least 24 h at room temperature. The retinas were then dissected and washed overnight in distilled water and incubated in 3% trypsin (Trypsin, Difco; 1:250 prepared in 0.1 M Tris, 0.1 M maleic acid, pH7.8 containing 0.2 M NaF) for approximately 1–1.5 h at 37°C. When digestion was completed, the very delicate and fragile retinal vessels were flattened by four radial cuts and mounted on glass slides for PAS and hematoxylin staining. Based on location and nuclear morphology of the cells, EC and PC were distinguished and counted. The nuclei of EC are oval or elongated and lie within the vessel wall along the axis of the capillary, while PC nuclei are small, spherical, stain densely, and generally have a protuberant position on the capillary wall. For quantification, slides were scanned by Aperio Digital Pathology Slide Scanner (ScanScope Model: CS, Aperio Technologies, Inc., Vista, CA) and images were captured at x40 using Aperio ImageScope software (version 12.2.2.5015; Leica Biosystems Imaging, Inc; Buffalo Grove, IL). The number of EC and PC was determined by counting the number of respective nuclei on captured images. The counting was performed in the mid-zone of the retina for at least six images from four quadrants of each retina, and retinas from at least 5 mice. The mean number of EC, PC, and their ratio are reported per image/field for each retina.

### Mice bodyweight assessment during development and OIR

Mice bodyweight was assessed by simply measuring their bodyweight (gram; gr) on a digital scale. The average mouse bodyweight is reported for each time point. During OIR, mice were weighed prior and after the course of 1, 25(OH)_2_D_3_ or saline administration at P12 and P17. Percentage of bodyweight gain was assessed by subtracting the bodyweight at P17 from P12 over the bodyweight at P12 ($(\frac{P17\;weight-P12\;weight}{P12\;weight})*100$).

### Determination of the rate vascular cell proliferation

We have previously shown that maximum vascular cell proliferation occurs in retinas from P14 mice [20]. Eyes were enucleated from P14 *Vdr* +/+ and *Vdr* -/- mice, fixed for 3 min in 4% paraformaldehyde, and stored in methanol at -20°C overnight. The retinas were then dissected and placed in PBS for 30 min, fixed in 3% paraformaldehyde for 30 min, and washed three times in PBS. The retinas were then transferred to new tubes, rinsed and blocked in blocking buffer (see above) for 24 h at room temperature. Next the retinas were incubated with Ki67 antibody (1:50 dilution, clone D3B5 catalog #:12075; Cell Signaling) in 2.5% BSA, 0.4% Triton X-100 and blocking buffer for 24 h at 4°C while rocking. Samples were washed 5 times in PBS, one time in 2.5% BSA, blocking buffer and 0.4% Triton X-100 (20 min at room temperature) and then rocked in 50% blocking buffer at room temperature for 30 min. The retinas were then incubated with the appropriate secondary antibody (1:500; Jackson ImmunoResearch Laboratories) in 2.5% BSA, blocking buffer and 0.4% Triton X-100 rocking for 2 h at room temperature. The samples were washed three times in PBS for 10 min and fixed in 3% paraformaldehyde for 30 min at room temperature. The retinas were then washed 3 times in PBS, transferred to new tubes and washed once more in PBS. The retinas were then incubated with Isolectin B4-FITC (1:100; Vector Labs) for 90 min and washed 3 times in PBS. The samples were mounted in mounting medium with DAPI (Southern Biotech). For quantification, the numbers of Ki67 positive cells on the blood vessels were determined per field (x400).

### Imaging of the hyaloid vasculature

Hyaloid vessels from 6-week-old mice were imaged using a Micron III indirect camera (Phoenix Research Labs, Pleasanton, CA). Mice were anesthetized using ketamine/xylazine and eyes were dilated with atropine. Fundus images were taken prior to an intraperitoneal injection of sodium fluorescein (100 mg/Kg) (10% soltution; Altaire Pharmaceuticals, Riverhead, NY) while the retina was in focus on the Micron III. Images were taken as the hyaloid vessels were filled with fluorescein.

### VEGF level measurements

The levels of VEGF were evaluated in retina extracts prepared from P15 *Vdr +/+*, *Vdr +/-*, and *Vdr -/-* mice using the Mouse VEGF Immunoassay Kit (R&D Systems, Minneapolis, MN). Briefly, retinas from each mouse were dissected and placed in 0.5 mL of PBS and stored at -80°C prior to analysis. VEGF levels were determined as recommended by the supplier.

### Statistical analysis

Statistical differences between groups were evaluated with the One-way ANOVA followed by Tukey’s multiple comparison test using GraphPad Prism version 5.04 for Windows (GraphPad Software, La Jolla, CA). Statistical Differences were confirmed with Bonferroni’s comparison of selected pairs of columns and student’s unpaired t-test (two-tailed). Mean ± standard deviation is shown. P< 0.05 is considered significant.

## Results

### The spreading of superficial layer of retinal vasculature is independent of Vdr expression

To assess the effect of *Vdr*-deficiency on retinal vascular development, we prepared retinal wholemounts from mice at various postnatal days and stained them to visualize the vasculature. In the majority of experiments, we used anti-collagen IV antibody (Col IV, stained red) to visualize the structure, organization, and regression of retinal blood vessels. Collagen IV is one of the major components of the basement membrane of retinal blood vessels. Here we assessed whether *Vdr*-deficiency influence the sprouting of the superficial layer of the retinal vasculature.

**Fig 1:**
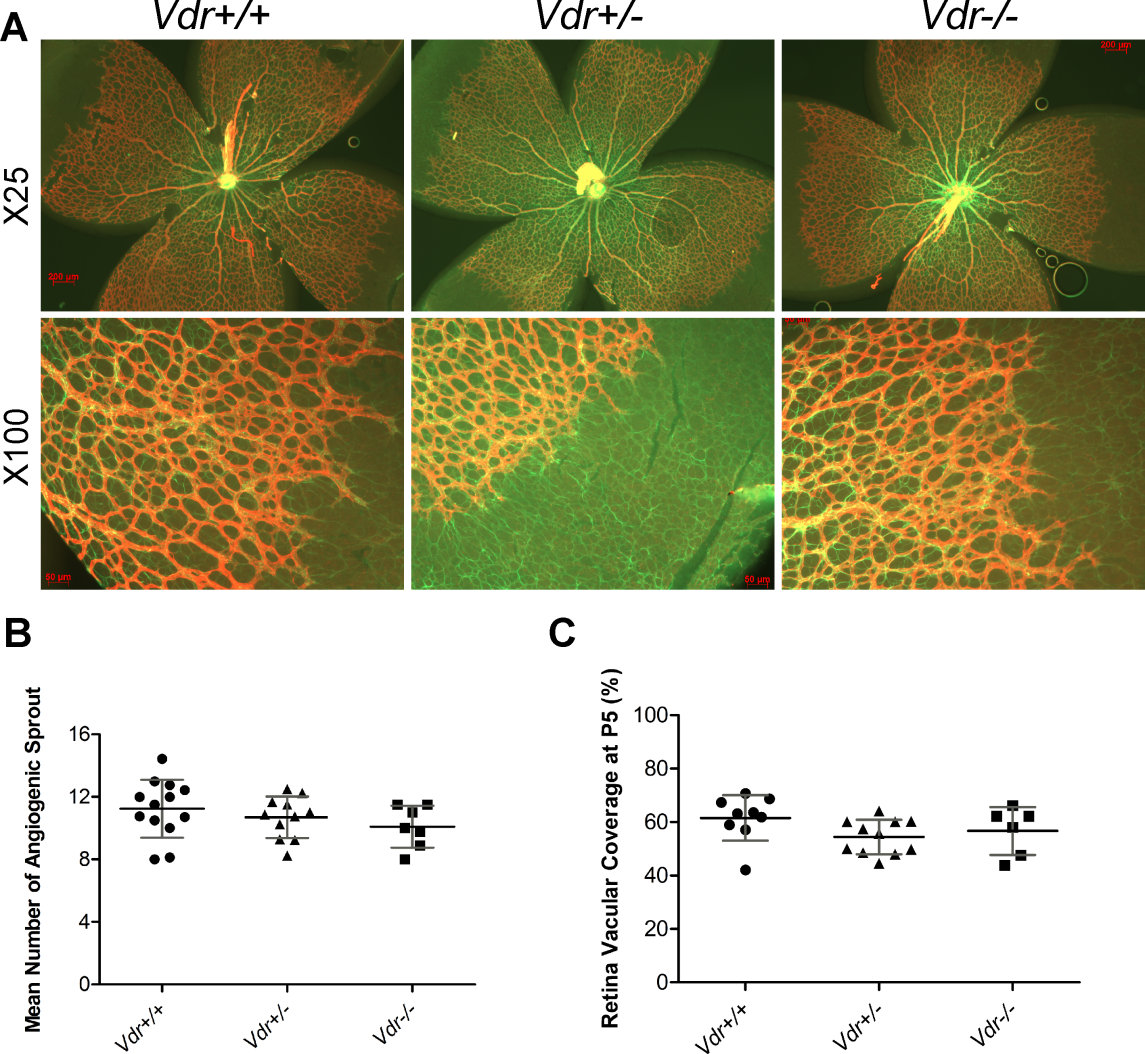
The development of superficial layer of retinal vasculature is independent of Vdr expression. (A) Demonstrates GFAP and Col IV stained retinal vessels prepared from postnatal day 5 (P5) Vdr +/+ and Vdr -/- mice. Please note similar expansion of astrocytes (green, GFAP) and progression of expanding vessels (red, Col IV). Scale bar = 200 μm for x25 and Scale bar = 50 μm for x100 images. (B) The mean number of angiogenic sprouts at the angiogenic fronts were quantified per field (x100) in each retina. (C) Coverage of retinal vasculature relative to total retina area were measured for each retina and is shown as a percentage. (n≥ 5; each point represents one mouse).

During early vascular development, retinal astrocytes (AC) lay out the primary scaffolding to guide retinal vascularization. Endothelial cells then follow this scaffolding to vascularize the retina. This is immediately followed by the recruitment of PC, which protect the EC and stabilize the newly formed blood vessels. Retinas from postnatal day five (P5) mice were stained for GFAP, an intermediate filament of AC, and flatmounted to assess the expansion of AC, and for Col IV to determine the rate of vascular EC expansion and the sprouting of endothelial tip cell density at the leading edge of expanding vasculature (Fig 1A). These results indicated no significant difference in the mean number of tip cell sprouts in *Vdr* -/- mice compared with *Vdr* +/- and *Vdr* +/+ littermates (Fig 1B). At this stage, retinal AC were reached the edge of retina and had covered the entire retina. Thus, the developing retinal vasculature expands at a similar rate in *Vdr* -/- and *Vdr* +/+ mice. We also measured the relative distance that the retinal vessels migrated from the optic nerve at P5. No significant differences were observed between *Vdr* +/+ and *Vdr* -/- littermates (Fig 1C).

### The organization of major blood vessels and development of primary retinal vascular plexus are not affected by Vdr-deficiency

**Fig 2:**
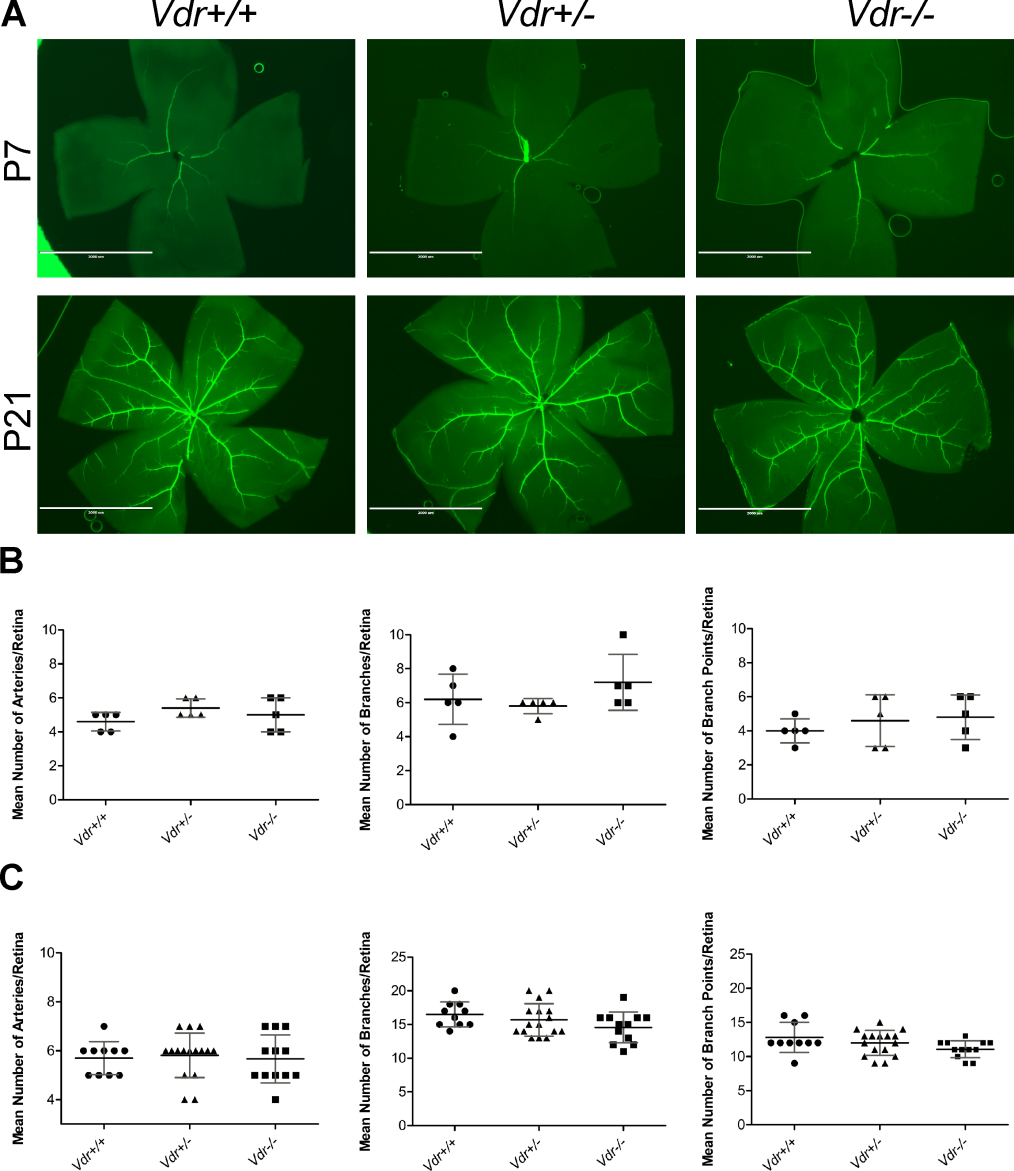
The organization of major blood vessels and development of primary retinal vascular plexus is not affected by Vdr-deficiency. (A) Retinas from P7 and P21 mice were wholemount stained with anti-α-smooth muscle actin and imaged at x12.5. Mean number of major arteries, branches, and branch points were quantified per retina and shown, respectively, in (B) for P7 and (C) for P21. (n≥ 5; each point represents one mice) Scale bar = 2,000 μm.

**Fig 3:**
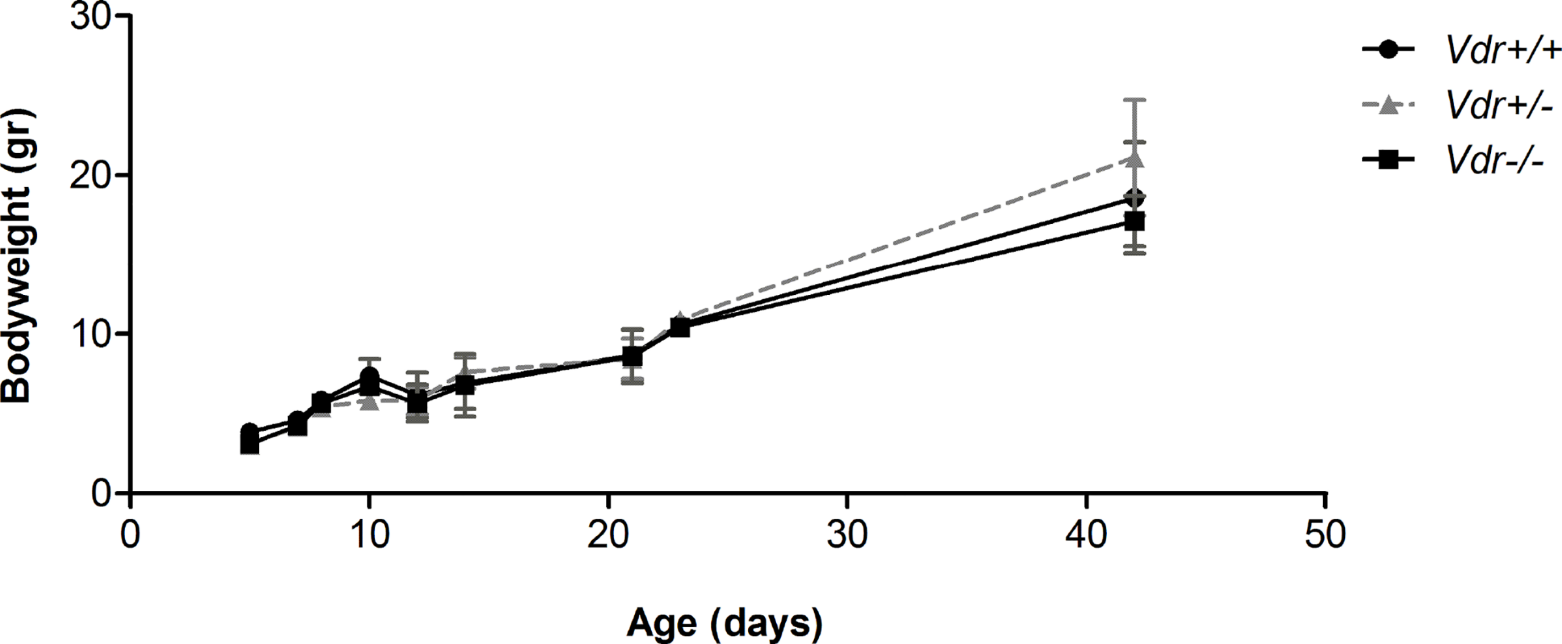
Vdr-deficiency minimally affected the mice bodyweight. Mice bodyweight were determined as detailed in Methods up to 6-weeks of age. **We observed no significant differences in postnatal mice bodyweight (gram) at each time point (n≥ 4)**.

We next examined whether *Vdr*-deficiency impacts the number of major blood vessels in the retina by wholemount staining with anti-α-smooth muscle actin (SMA). The SMA is mainly expressed in smooth muscle cells, which cover the major arteries but not the capillaries [6]. We examined retinas from P7 mice, when the formation of superficial layer of blood vessels is nearly complete, and P21 mice when the formation of primary vascular plexus is completed (Fig 2A). At P7, we observed a similar mean number of retinal arteries in *Vdr* +/+ (4.6 ± 0.54) and *Vdr* -/- (5 ± 1.00) mice. The mean number of major arteries, branches, and branch points were also similar between *Vdr* -/- mice and their *Vdr* +/- and *Vdr* +/+ littermates at P7 and P21 (Fig 2B and 2C). Thus, our results indicated minimal association between *Vdr*-deficiency and organization of major blood vessels comparing *Vdr* -/- mice with their *Vdr* +/+ and *Vdr* +/- littermates. We also observed similar structure and density of the retinal vasculature at P5, P8, P10, P14, and P21 in wholemounts and P8 and P10 in frozen sections, and similar rates of proliferations determined by Ki67 staining (Figure A in S1 File). These results are also consistent with the minimal differences observed in the bodyweights among these mice (Fig 3). Furthermore, examination of hyaloid vessels regression after 6-weeks of age revealed no differences between *Vdr +/+* and *Vdr -/-* mice (Figure B in S1 File).

### Decreased vascular cell density and EC/PC ratios in Vdr -/- mice

**Fig 4:**
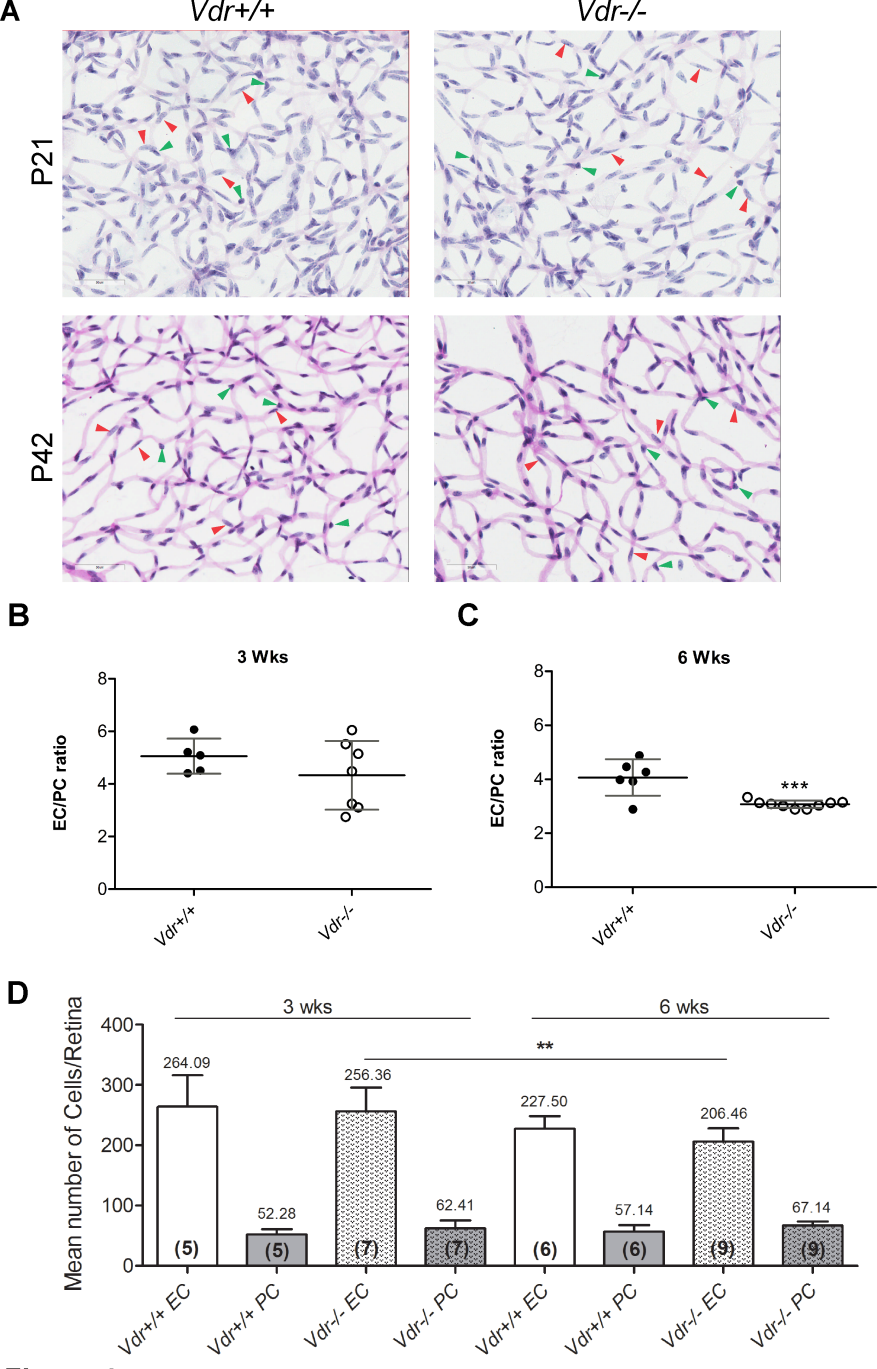
Altered vascular cell density and EC/PC ratios in Vdr -/- mice. Retinas from P21 and P42 mice were prepared by trypsin digest and H&E/PAS staining. Slides were then scanned and images captured at x400. (A) Representative images are shown; Scale bar = 50 μm. Number of EC (red arrow head) and PC (green arrow head) were counted for at least 6 images per mice, and EC/PC ratio calculated for P21 (B) and for P42 (C) mice; (***P = 0.0008). (D) The quantitative assessment of this data, and the number of EC and PC along with the number of retinas counted in each group in parentheses, are shown. (n≥ 5; **P = 0.0043).

To compare retinal vascular densities in *Vdr* +/+ and *Vdr* -/- mice, we prepared retinal trypsin digests and determined EC/PC ratios as well as their densities. In wholemount retinal digests, the EC nuclei occur within the vessel wall, are large, oval, weakly stained, and protrude luminally. Pericytes nuclei, are darkly stained, small, round, and protrude laterally from the vessel wall. Fig 4A shows trypsin-digest preparations from P21 mice, when formation of primary vasculature is completed, and P42 mice, after completion of vascular maturation. Our data demonstrated no significant changes in EC/PC ratio in retinal vasculature of P21 *Vdr* -/- mice compared with *Vdr* +/+ mice (Fig 4B). However, a significant decrease in the EC/PC ratio was observed in retinal vasculature of 6-week-old *Vdr* -/- mice compared with *Vdr* +/+ mice (Fig 4C). Loss of EC, which normally occurs during maturation of developing retinal vasculature, was also observed here regardless of VDR expression. In contrast, the loss of PC during normal maturation of developing retinal vasculature is minimal [7, 20, 21]. Thus, the significant decrease in EC/PC ratio of retinal vasculature from 6-week-old *Vdr* -/- mice was attributed to the presence of increased number of PC, which continued to accumulate in the absence of VDR, and decreased number of EC (Fig 4D). Collectively, our data suggest that expression of VDR is essential for appropriate maturation of the retinal vasculature, by halting the promigratory and proliferative phenotype of pericytes and enhancing the survival of EC following the formation of retinal primary vascular plexus.

### Retinal neovascularization during OIR is independent of Vdr expression

Oxygen induced ischemic retinopathy in mice allows to study the various phases of ROP, the hyperoxia mediated vessel obliteration and ischemia-mediated neovascularization [13, 14]. In this model, P7 pups and their mother are exposed to a cycle of hyperoxia (75% oxygen) and normoxia/hypoxia (20% oxygen) for five days. High oxygen exposure results in downregulation of proangiogenic factors that prevent further development of retinal vasculature and promotes loss of existing blood vessels (vessel obliteration). When mice are exposed to room air (normoxia), the under vascularized retina becomes ischemic and formation of new abnormal blood vessels (neovascularization) will initiate. These new abnormal blood vessels grow from the retina into the vitreous, and form vascular tufts. Mice were sacrificed at desired time points and, areas of vessel obliteration and degree of neovascularization were assessed.

**Fig 5:**
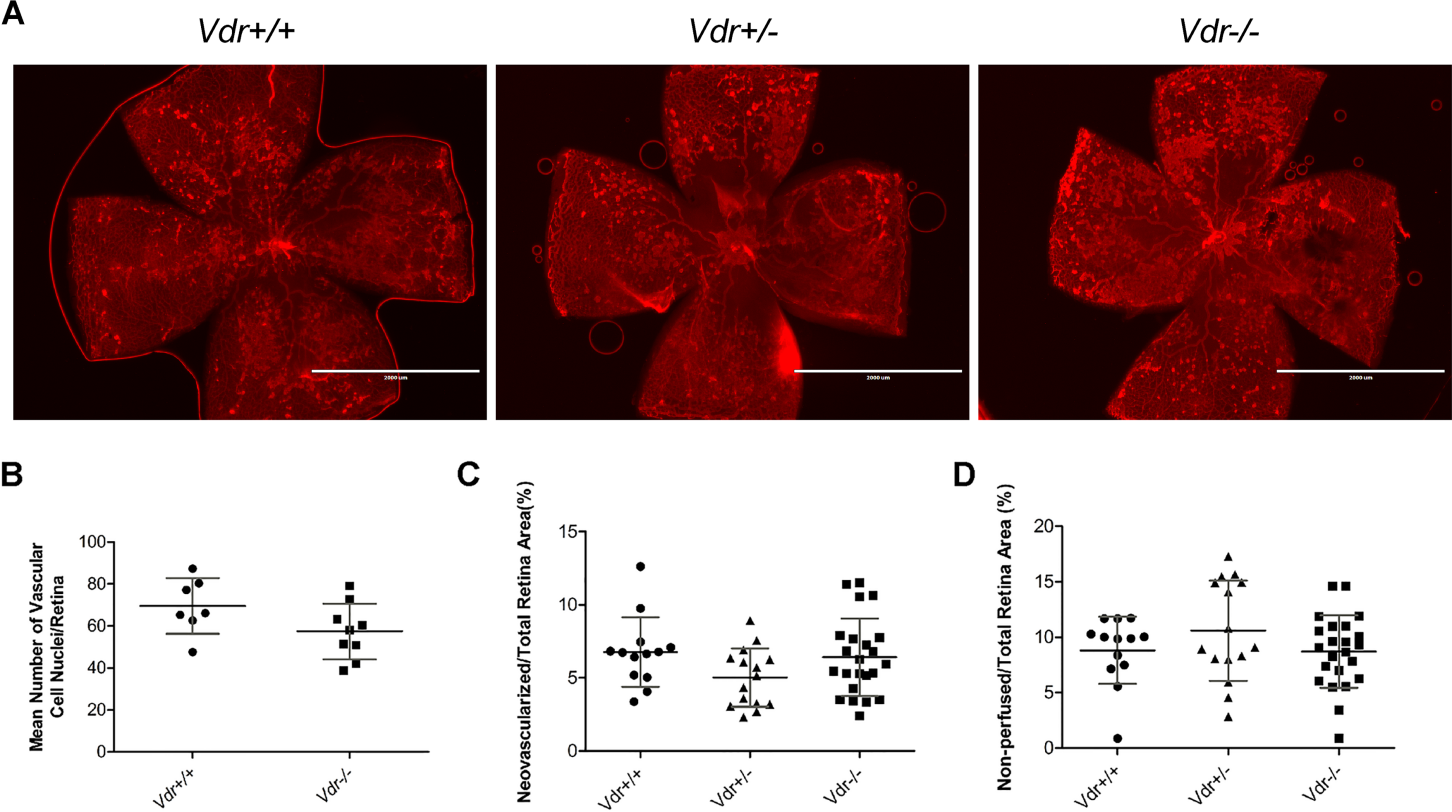
Similar degree of ischemia-driven retinal neovascularization in Vdr+/+ and Vdr-/- during OIR. (A) Representative images (x20) of wholemount retinal neovascularization isolated from P17 mice exposed to a cycle of hyperoxia and room air (OIR) and stained with collagen IV. Retinas from Vdr +/+, Vdr +/-, and Vdr -/- littermates were wholemount stained with anti-collagen IV to visualize the vasculature. Scale bar = 2,000 μm. Quantitative assessment of the neovascularization (histological evaluation and quantitative analysis of images) and area of vessel obliteration are shown in (B), (C), and (D) respectively. (n≥ 7; each point represents one mice).

Representative images (x20) of wholemount retinas stained with Col IV from *Vdr* +/+, *Vdr* +/-, and *Vdr* -/- mice (Fig 5A) demonstrated similar hyperoxia-mediated retinal vessel obliteration (Fig 5D). Retinal neovascularization was assessed using two different methods with similar results (Fig 5B and 5C). No significant differences in the area of vessel obliteration or degree of neovascularization was observed in *Vdr +/+* mice compare to their *Vdr -/-* littermates. Thus, retinal neovascularization during OIR is independent of VDR expression.

### 1, 25(OH)_2_D_3_-mediated inhibition of retinal neovascularization requires Vdr expression

**Fig 6:**
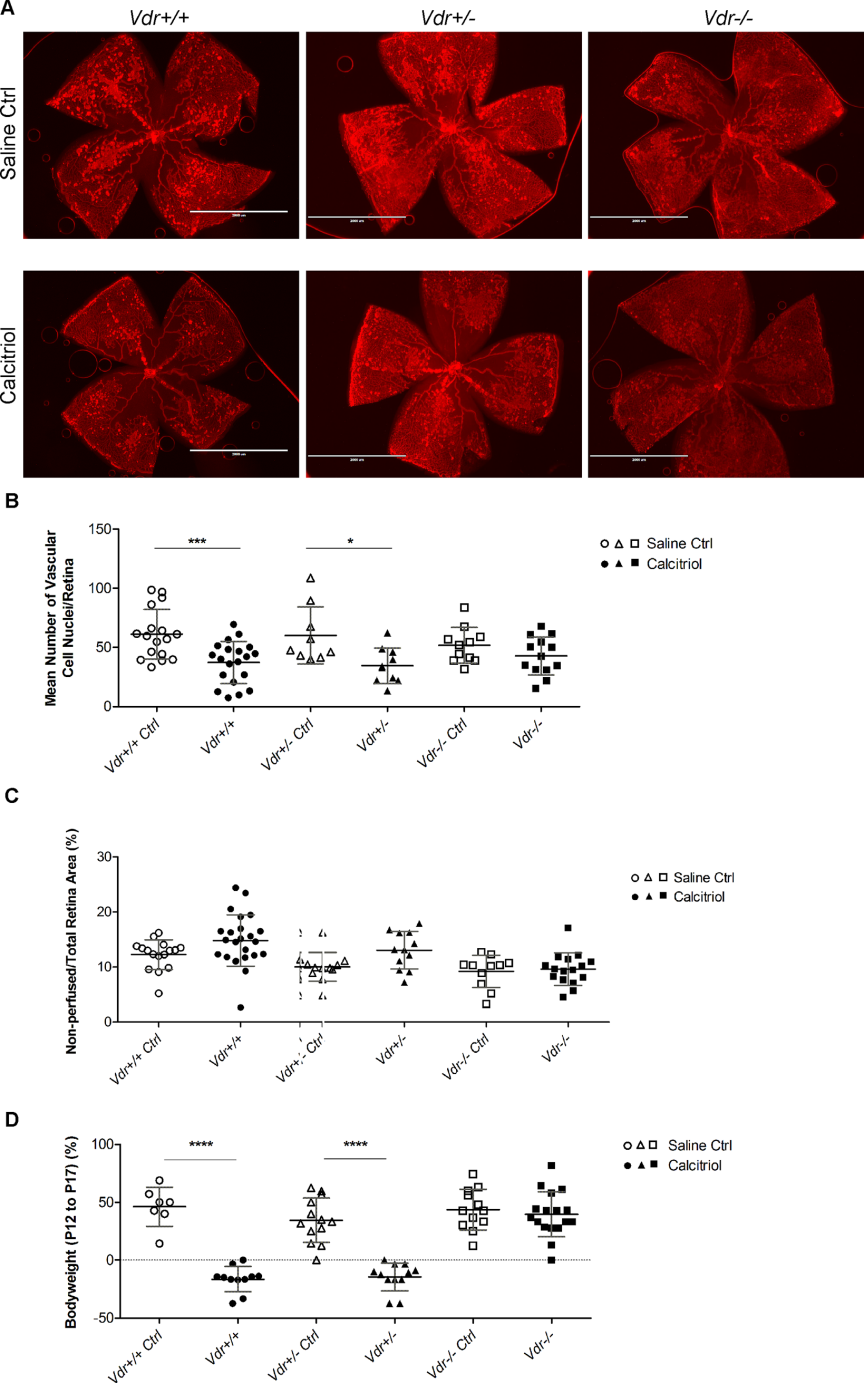
*Vdr* expression is required for significant inhibition of retinal neovascularization by1, 25(OH)_2_D_3_. (A) Representative images (x20) of collagen IV stained wholemount retinal neovascularization from P17 OIR with and without 1, 25(OH)_2_D_3_ treatment from *Vdr* +/+, *Vdr* +/-, and *Vdr* -/- mice. Scale bar = 2,000 μm. Quantitative assessment of the neovascularization (histological evaluation) and area of vessel obliteration from these groups are shown in (B) and (C), respectively; (***P = 0.0006, *P = 0.0121). (D) The evaluated mice bodyweight (gr) comparison from the above groups is shown; (****P<0.0001). (n≥ 7; each point represents one mouse).

Previous studies from our laboratory demonstrated that 1, 25(OH)_2_D_3_ is a potent inhibitor of retinal neovascularization during OIR [14]. We next determined whether this inhibition of neovascularization is dependent on VDR expression. We compared the effects of 1, 25(OH)_2_D_3_ on retinal neovascularization in *Vdr* +/+, *Vdr* +/-, and *Vdr* -/- during OIR (Fig 6A). Since room air/normoxia cycle during OIR occurs from P12 to P17, 1, 25(OH)_2_D_3_ was administered by daily intraperitoneal injections during this time and the degree of neovascularization was assessed at P17. Using the quantitative image analysis and/or counting the number of nuclei in neovascular tufts on the vitreous side, our results demonstrated that the significant inhibition of neovascularization by 1, 25(OH)_2_D_3_ was dependent on VDR expression (Fig 6B and Figure D in S1 File). 1, 25(OH)_2_D_3_ treatment had no significant impact on the area of vessel obliteration observed between groups (Fig 6C). In addition, 1, 25(OH)_2_D_3_ mediated bodyweight loss, which is a systemic side effect of 1, 25(OH)_2_D_3_ treatment was not observed in *Vdr -/-* mice (Fig 6D). Thus, although degree of retinal neovascularization is independent of *Vdr* expression during OIR (as shown in Fig 5B and 5C; non-treated mice), the significant inhibition of retinal neovascularization by 1, 25(OH)_2_D_3_ is *Vdr* dependent (as shown in Fig 6B and Figure D in S1 File; 1, 25(OH)_2_D_3_ treated mice).

## Discussion

Vitamin D is one of the natural compounds, which in its hormonal and active form (Calcitriol or 1, 25(OH)_2_D_3_) inhibits the development, growth, and progression of a variety of cancers and eye diseases. This inhibitory effect is mainly mediated through VDR and its direct effect on gene expression [14, 22–26]. However, based on observed rapid responses after vitamin D treatment, potential non-genomic functions of vitamin D are also suggested [27]. These functions could be mediated directly by VDR, as well as those by other membrane-associated receptors [27–30]. Thus, vitamin D’s action could be mediated through different receptors and pathways. Here we investigated the impact of VDR expression on postnatal retinal vascular development and neovascularization during OIR. Based on reduced vascularity and growth of tumors treated with vitamin D and its analogs [31, 32], it was proposed that vitamin D and its analogs have anti-angiogenic activity, and tumor vasculature might be a target [25]. Our previous studies showed significant attenuation of retinal neovascularization by 1, 25(OH)_2_D_3_ during OIR [14]. However, the role VDR expression plays in retinal vascular development and neovascularization during OIR and its inhibition by 1, 25(OH)_2_D_3_ remained unknown.

To further understand VDR mechanisms of action, transgenic mice lacking a functional Vdr expression have been generated. *Vdr-*deficient (*Vdr* -/-) mice are viable with a normal phenotype at birth and they do not exhibit any developmental and growth defects, especially, before weaning [33, 34]. VDR function has been investigated in developmental studies [35–37] as well as its association with various diseases [3, 38–40], cancer [41–48], and carcinogen-induced tumorigenesis [49]. These studies indicated that *Vdr-*deficiency impairs inner ear development [37] and its expression is essential for heart development in zebrafish [36]. Mice deficient in VDR also showed accelerated mammary gland development during pregnancy with delayed post-lactation involution, perhaps as a result of enhanced angiogenesis during development and failure in proper vessel regression during involution [35]. These observations are consistent with our findings that VDR expression, especially in perivascular supporting cells, has a significant impact on the maturation of developing blood vessels by promoting the quiescence and differentiated phenotype of these cells. Here we showed that the mice bodyweight was similar between *Vdr* +/+, *Vdr* +/-, and *Vdr* -/- during early postnatal development up to 6-weeks of age. Thus, it is highly unlikely that the changes we report here in *Vdr* -/- mice are associated with adverse health issues in these mice noted as they get older, which could be simply remedied by appropriate diet. However, some of the cardiovascular abnormalities associated with vitamin D deficiency is only revered by addition of vitamin D supplement and not with rescue diet or calcium normalization [50].

The postnatal development of retinal vasculature permits to study all aspects of vascular development after birth. We next examined normal postnatal retinal vascular development and retinal neovascularization during OIR in *Vdr* +/+, *Vdr* +/-and *Vdr* -/- mice at different time points. *Vdr* +/+ and *Vdr* -/- mice exhibited a very similar pattern of vascular development and vascular density up to three weeks of age, when the formation of primary retinal vascular plexus is completed but not matured. However, the EC to PC ratio of *Vdr* -/- mice decreased significantly by 6-weeks of age, after remodeling and pruning, and maturation of retinal vasculature. This decrease in the EC/PC ratio was mainly attributed to the presence of increased number of PC in *Vdr* -/- mice. The number of EC decreased as occurs normally during this process. However, this decrease in the number of EC was further impacted by *Vdr*-deficiency perhaps as a result of reduced VEGF levels produced by *Vdr* deficient PC resulting in less protection. Thus, changes in PC density and maturation may have a significant impact on vascular function [51]. Alterations in EC and PC ratio have been observed under a variety of conditions and disease states including aging, diabetic retinopathy, cancer, hyperglycemia, multiple sclerosis, and during development [51–58]. Chen et al. recently reported blood vessels in the periphery of tumors have higher PC coverage and are more resistant to vascular disrupting agents, which contribute to treatment failure and diseases recurrence [59]. How changes in *Vdr*-deficient PC characteristics and increased numbers may impact retinal vascular function and susceptibility to various insults awaits further investigation.

The increase in the number of PC could be attributed, at least in part, to our observation that 1, 25(OH)_2_D_3_ inhibits the proliferation of *Vdr* +/+ PC in culture [5], as previously shown in smooth muscle cells (SMC) [60, 61]. Thus, the increase in PC number in *Vdr* -/- could be associated with lack of response to endogenous vitamin D signal and maturation of retinal vasculature. Incubation of vascular SMC with vitamin D results in increased production of VEGF [62]. This increase in VEGF is demonstrated to have a negative effect on proliferation of perivascular supporting cells through promotion of heterodimerization of PDGF-Rβ and VEGF-R2 [63]. The heterodimerization of these receptors attenuate signaling through these receptors, by their respective ligands, and promoting the coverage and maturation of developing blood vessels [63]. We have also observed increased production of VEGF in retinal PC incubated with vitamin D, as previously shown in SMC [64]. Thus, we propose that 1, 25(OH)_2_D_3_ acting through VDR inhibits proangiogenic activity of PC by promoting their quiescence and stabilizing the newly formed blood vessels though increased VEGF production. This also may promote the survival of EC in mature blood vessels, which depend on the VEGF produced by PC [65].

The increase in VEGF level by 1, 25(OH)_2_D_3_ occurs through direct interactions between VDR, as a transcription factor, and VEGF promoter in vascular SMC [66]. Thus, in the absence of VDR, we propose the reduced levels of VEGF produced by PC could be responsible for their increased number, and perhaps reduced number of EC whose survival is dependent on VEGF production. Although the changes in VEGF level in the retinas of *Vdr* -/- mice appeared not to be significantly different from *Vdr* +/+ mice (Figure C in S1 File), this difference may be masked due to various production of VEGF by multiple cells in the retina including EC. The direct impact of VDR expression on production of VEGF by PC incubated with vitamin D awaits evaluation of VEGF levels in *Vdr +/+ and Vdr -/-* PC. Together, our data show that the primary postnatal development of retinal vasculature is independent of VDR expression. However, vascular density and ratio of EC and PC is affected by VDR expression, at 6-weeks of age. These observations suggest an important role for VDR expression during remodeling and maturation phase of the developing retinal vasculature through PC quiescence. This is very similar to the impact of other angioinhibitory factors we previously examined during postnatal retinal vascular development including thrombospondin-1 (TSP1) and pigment epithelium derived factor (PEDF). We showed the deficiency in these genes minimally affected formation of primary retinal vascular plexus [20, 67]. We proposed that this is contributed to the overwhelming active role of proangiogenic factors during active angiogenesis. However, when proangiogenic activity diminishes in order to establish the homeostatic state, the antiangiogenic factors could take an active role in eliminating excess vasculature until a state of homeostasis is established, as occurs during pruning and remodeling, and maturation of developing vasculature.

We next examined the impact of VDR expression on retinal vessel obliteration and neovascularization during OIR. We observed no significant differences in degree of vessel-obliteration in *Vdr +/+* compare to *Vdr -/-* mice. In addition, the *Vdr* -/- mice exhibited a similar rate of retinal neovascularization compared with *Vdr* +/+ mice (non-treated). Thus, VDR expression minimally impacts retinal vascular responses during OIR. This is consistent with our previous observation that deficiency of other angioinhibitory factors, namely TSP1 and PEDF, minimally affect neovascularization during OIR [20, 67]. Again, we propose that this is mainly attributed to the active and dominant role of proangiogenic factors during active angiogenesis. It is only when antiangiogenic molecules are in excess (exogenously added) or the level of proangiogenic factors drop below a threshold (excess endogenous level) when inhibitors of angiogenesis prevent angiogenesis, as occurs during pruning and remodeling, and maturation of newly formed vessels [67]. We previously showed exogenous 1, 25(OH)_2_D_3_ inhibited retinal neovascularization during OIR [14]. Here we showed, 1, 25(OH)_2_D_3_ failed to significantly inhibit retinal neovascularization in *Vdr* -/- mice compared with *Vdr* +/+ mice during OIR (in 1, 25(OH)_2_D_3_ treated mice). Thus, a significant anti-angiogenic activity of 1, 25(OH)_2_D_3_ is mediated through VDR. However, whether VDR-independent mechanisms may also contribute to vitamin D-mediated inhibition of angiogenesis awaits carful cell autonomous characterization of vitamin D action on retinal vascular cells. A potential systemic side effect of 1, 25(OH)_2_D_3_ treatment is hypercalcemia and lack of bodyweight gain [68, 69]. Interestingly, 1, 25(OH)_2_D_3_ mediated weight loss was not observed in *Vdr* -/- mice compared with *Vdr* +/+ mice during OIR (1,25(OH)_2_D_3_ treated mice).

In summary, our results indicate that VDR expression is an important modulator of vascular development, especially during late stages, where it promotes the quiescence of perivascular supporting cells and maturation of blood vessels. Although, pathological retinal neovascularization during OIR was independent of VDR expression, the significant inhibition of retinal neovascularization by 1, 25(OH)_2_D_3_ was dependent on expression of VDR. Identification and understanding the 1, 25(OH)_2_D_3_ mechanisms of action during biological and pathological conditions, and the signaling pathways involved will improve our knowledge regarding its therapeutic use.

## Supporting information

S1 File Supplementary figures. Supplementary Figures_NJ.docx.
(DOCX)
