# Supplementary material for: Vitamin D receptor expression is essential during retinal vascular development and attenuation of neovascularization by 1, 25(OH)2D3
Source: PLoS One. 2017 Dec 22;12(12):e0190131. doi: 10.1371/journal.pone.0190131 (PMC5741250; doi:10.1371/journal.pone.0190131)
Supplement: S1 File — Supplementary Figures_NJ.docx. (DOCX) [file pone.0190131.s001.docx]

# **Supplementary Figures**


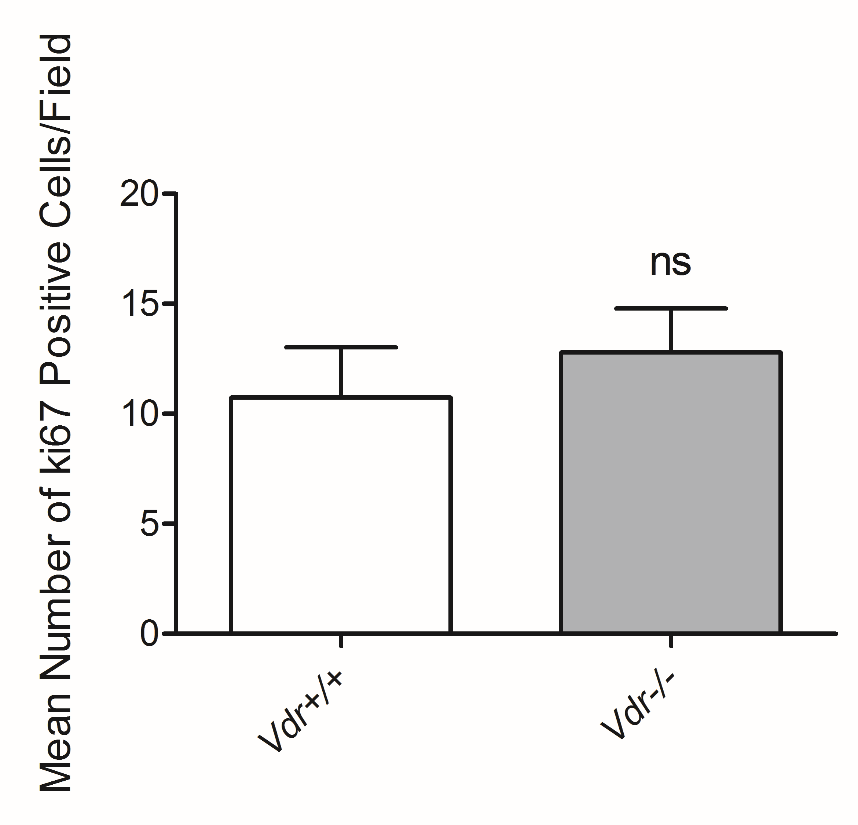


## **Fig A: Proliferating cells of the retina at postnatal day 14 (P14) is not affected by *Vdr* deficiency.**

Proliferating cells in retinas from p14 *Vdr +/+* and *Vdr -/-* mouse were assessed by anti-Ki67 staining. The data in each bar is the mean number of Ki67 positive cells counted per field (x400). Please note no significant difference was observed between the two groups (P=0.38).


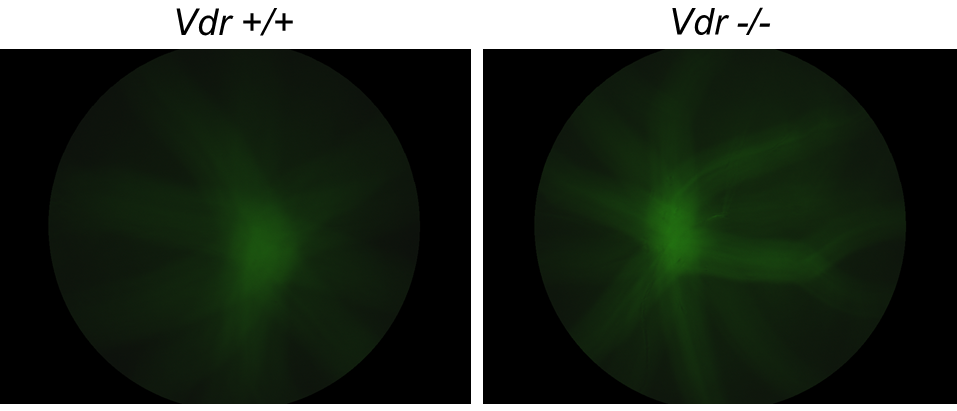


## **Fig B: Regression of hyaloid vasculature is independent of Vdr expression.**

Hyaloid vessels of 6-week-old mice were imaged using a Micron III indirect camera. Fundus images were taken prior to an intraperitoneal injection of sodium fluorescein 10% solution (100 mg/Kg). While the retina was in focus on the Micron III, images were taken as the hyaloid vessels filled with fluorescein. Please note the absence of hyaloid blood vessels in *Vdr* +/+ and *Vdr* +/- mice. Hyaloid vessels were similarly regressed by six weeks of age (n= 5).

## **
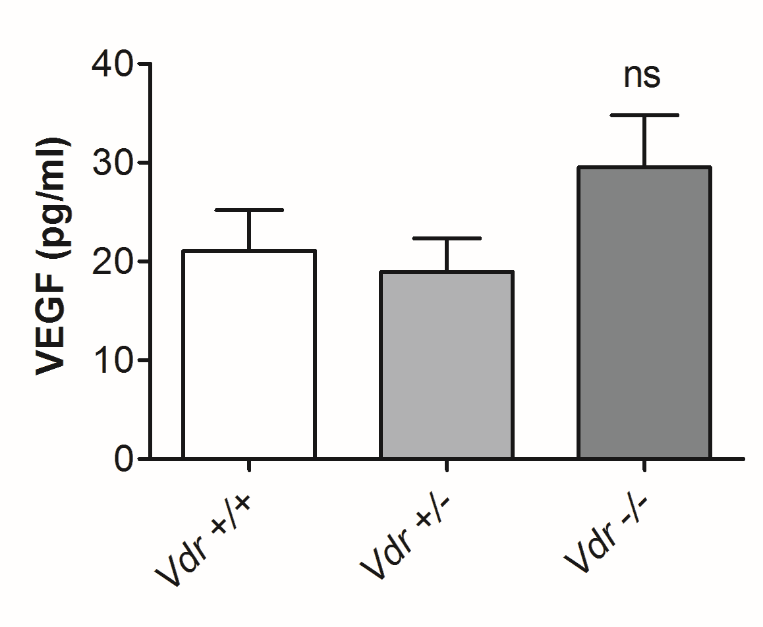
**

## **Fig C: The retinal VEGF levels were independent of Vdr expression.**

VEGF level was assessed by ELISA in the retinas from *Vdr +/+*, *Vdr +/-*, and *Vdr -/-*. No significant changes was observed between the groups using one way ANOVA (P=0.083; n=3).


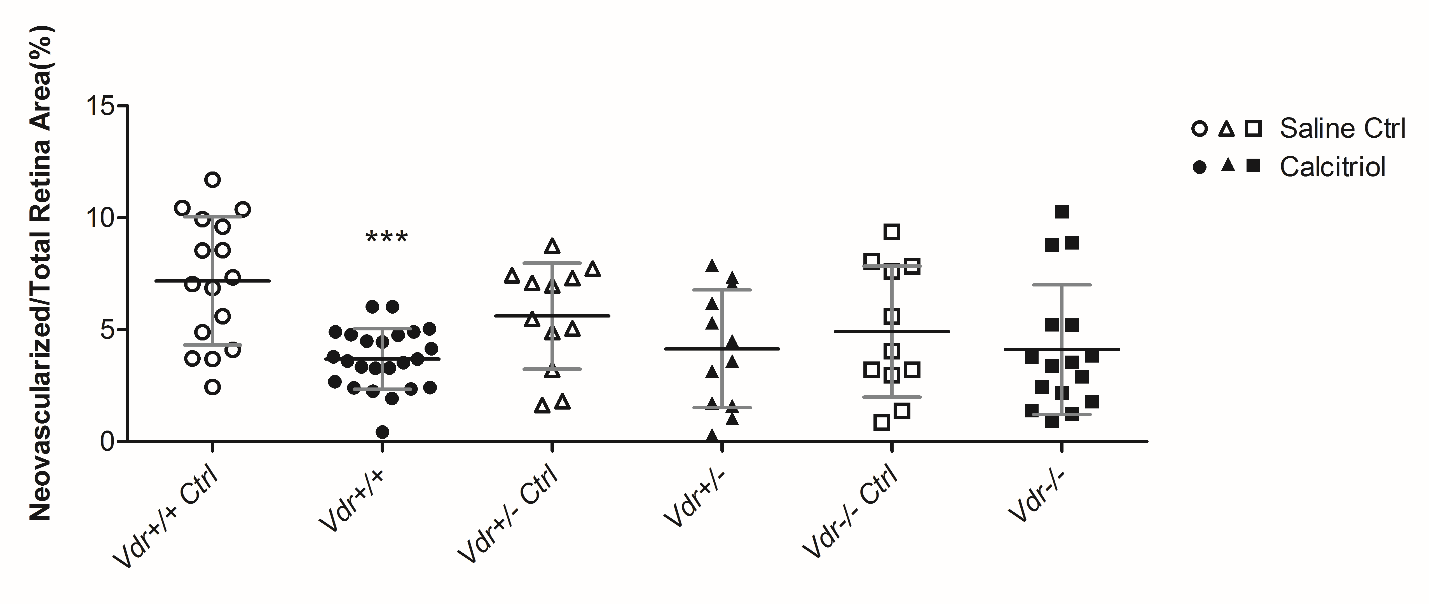


## **Fig D: *Vdr* Expression is required for significant inhibition of retinal neovascularization by 1, 25(OH)_2_D_3_.**

Quantitative analysis of images of wholemount retinal neovascularization isolated from P17 mice exposed to a cycle of hyperoxia and room air (OIR), which received 1, 25(OH)_2_D_3_ from P12 to P16. (n*≥*11; each point represents one mice)
